# Three weeks of respiratory muscle endurance training improve the O_2_ cost of walking and exercise tolerance in obese adolescents

**DOI:** 10.14814/phy2.13888

**Published:** 2018-10-22

**Authors:** Hailu K. Alemayehu, Desy Salvadego, Miriam Isola, Gabriella Tringali, Roberta De Micheli, Mara Caccavale, Alessandro Sartorio, Bruno Grassi

**Affiliations:** ^1^ Department of Medicine University of Udine Udine Italy; ^2^ Istituto Auxologico Italiano IRCCS Experimental Laboratory for Auxo‐endocrinological Research Milan and Piancavallo (VB) Italy; ^3^ Division of Metabolic Diseases and Auxology Istituto Auxologico Italiano IRCCS Piancavallo (VB) Italy; ^4^ Institute of Bioimaging and Molecular Physiology National Research Council Milan Italy

**Keywords:** Obesity, respiratory muscle endurance training, O_2_ cost of walking, O_2_ cost of breathing

## Abstract

Obese adolescents (OB) have an increased O_2_ cost of exercise, attributable in part to an increased O_2_ cost of breathing. In a previous work a short (3‐week) program of respiratory muscle endurance training (RMET) slightly reduced in OB the O_2_ cost of high‐intensity cycling and improved exercise tolerance. We hypothesized that during treadmill walking the effects of RMET would be more pronounced than those observed during cycling. Sixteen OB (age 16.0 ± 0.8 years; body mass [BM] 127.7 ± 14.2 kg; body mass index 40.7 ± 4.0 kg/m^2^) underwent to 3‐week RMET (*n *=* *8) superimposed to a multidisciplinary BM reduction program, or (CTRL, *n *=* *8) only to the latter. Heart rate (HR) and pulmonary O_2_ uptake ($\dot{V}$O_2_) were measured during incremental exercise and 12‐min constant work rate (CWR) walking at 60% (moderate‐intensity, MOD) and 120% (heavy‐intensity, HEAVY) of the gas exchange threshold (GET). The O_2_ cost of walking (aerobic energy expenditure per unit of covered distance) was calculated as $\dot{V}$O_2_/velocity. BM decreased (~4–5 kg) both in CTRL and in RMET. $\dot{V}$O_2_peak and GET were not affected by both interventions; the time to exhaustion increased following RMET. During MOD and HEAVY RMET decreased $\dot{V}$O_2,_ the O_2_ cost of walking (MOD: 0.130 ± 0.033 mL/kg/m [before] vs. 0.109 ± 0.027 [after], *P *=* *0.03; HEAVY: 0.196 ± 0.031 [before] vs. 0.180 ± 0.025 [after], *P *=* *0.02), HR and rates of perceived exertion; no significant changes were observed in CTRL. In OB a short RMET program lowered the O_2_ cost of MOD and HEAVY walking and improved exercise tolerance. RMET could represent a useful adjunct in the control of obesity.

## Introduction

Obese patients have a higher O_2_ cost of exercise (Wasserman and Whipp 1975; Lafortuna et al. 2008; Salvadego et al. 2010), which negatively affects exercise tolerance (Grassi et al. 2015) and is at least in part attributable to a higher O_2_ cost of breathing (Kress et al. 1999; Salvadego et al. 2015, 2017). Obesity has indeed a profound effect on the physiology of breathing (Luce 1980; Koenig 2001). In obese subjects resting and exercise tidal breathing occur at low operational lung volumes, thereby increasing the prevalence and severity of expiratory flow limitation and the resistive load on the respiratory system (Littleton 2012). The reduced chest wall compliance, attributable to the excess fat mass (FM) on the respiratory wall, and the increased work to be performed against abdominal fat and viscera further increase the work of breathing (Kress et al. 1999; Littleton 2012). In association with respiratory muscle inefficiency, the increased work of breathing determines a substantially higher O_2_ cost of breathing (Koenig 2001), contributing to the higher O_2_ cost of exercise (Wasserman and Whipp 1975; Salvadego D et al. 2010; Salvadego et al. 2015, 2017). This is exacerbated by the higher pulmonary ventilation at the same work rate observed in obese patients versus normal weight controls (Cherniack 1959; Kress et al. 1999), possibly leading to exertional dyspnea (Scano et al. 2009).

The increased O_2_ cost of breathing could entail a “competition” between respiratory and locomotor muscles for the finite available O_2_ (Harms et al. 2000), leading to fatigue and premature exhaustion. This would contribute to the inactivity which represents one of the main causes of obesity, impeding the increased level of physical activity which is one of the cornerstones of the treatment of the disease.

In order to interrupt this vicious circle, we recently followed two approaches, attempting to relieve the respiratory limitation in obese adolescents performing cycling exercise. In the first study (Salvadego et al. 2015) respiratory muscles (RM) were acutely unloaded by normoxic helium‐O_2_ breathing. Helium [He] has indeed a lower density compared to nitrogen, and thereby He‐O_2_ breathing requires less respiratory muscle work than air breathing. The intervention reduced the O_2_ cost of cycling and the perception of fatigue during moderate‐ and heavy‐intensity CWR exercise. In the second study (Salvadego et al. 2017) a standardized program of respiratory muscle endurance training (RMET) (Spengler and Boutellier 2000; Sheel 2002; Rigamonti et al. 2014) was superimposed on a standard multidisciplinary BM reduction program; the intervention slightly decreased perceived exertion and O_2_ cost of cycling during heavy‐, but not during moderate‐intensity exercise, and improved peak exercise capacity.

The aim of the present study was to evaluate the hypothesis that the RMET effects on the O_2_ cost of exercise and exercise tolerance would be more pronounced during walking on a treadmill compared to the effects described during cycling (Salvadego et al. 2017), and/or could be identified also during moderate‐intensity exercise. Since the mechanical pattern of walking entails the cyclical elevation and acceleration of body center of mass at every step, treadmill exercise is a relatively costly type of locomotion when compared to cycling (Lafortuna et al. 2008). Obese patients should be more heavily penalized (vs. normal weight controls) during weight bearing activities like walking or running compared to cycling. The much larger muscle mass in action during walking or running than during cycling would aggravate the exertional dyspnea and could enhance the competition between respiratory and locomotor muscles for the available O_2_, thereby determining a larger “signal” on which to intervene with RMET.

## Methods

### Subjects

Sixteen male obese patients (age 15–18 years; Tanner stage 4–5), hospitalized for a multidisciplinary BM reduction program, were admitted to the study. Patients were randomly assigned to RMET (*n *=* *8; 16.5 ± 0.9 years; BM 130.5 ± 18.4 kg) or to a control (CTRL) group, (*n *=* *8; 15.5 ± 0.9 years; BM 124.9 ± 10.0 kg). The RMET group followed a specific program of RMET in addition to the standard multidisciplinary BM reduction program (see below). The CTRL group underwent only the standard BM reduction program.

Participants’ parents provided signed consent statements, after being fully advised about the purposes and testing procedures of the investigation, which were approved by the ethics committee of the Italian Institute for Auxology, Milan, Italy. All procedures were in accordance with the recommendations set forth in the Helsinki Declaration (World Medical Association. 2001).

Body mass index (BMI) was calculated as BM divided by height^2^, expressed in (kg/m^2^). The standard deviation score (SDS) of BMI was calculated by applying the LMS method (based upon the skewness [L], the median [M], and the coefficient of variation [S] of the measurements as a function of age) to Italian reference values for children and adolescents (Cacciari et al. 2006).

Body composition was determined by bioelectrical impedance (Human‐IM Scan, DS‐Medigroup, Milan, Italy). FM and fat free mass (FFM) were expressed as kg and as a percentage of BM. All examinations were performed by the same investigator before and after the 3‐week intervention period (see below).

Inclusion criteria were: (1) BMI > 97th percentile for age and sex, using Italian growth charts (Cacciari et al. 2006); (2) no involvement in structured physical activity programs (regular activity more than 120 min/week) during the 8 months preceding the study; (3) absence of signs or symptoms of diabetes or of any major cardiovascular, respiratory or orthopedic disease contraindicating or significantly interfering with the tests.

### BM reduction intervention

The patients were admitted as in‐patients (Division of Auxology, Italian Institute for Auxology, Piancavallo, Italy) for a 3‐week in‐hospital multidisciplinary BM reduction intervention (Salvadego et al. 2017), involving the following: (1) Moderate energy restriction, with a personalized diet entailing an energy intake ~500 kcal lower than the measured resting energy expenditure. Diet composition was formulated according to the Italian recommended daily allowances (Società Italiana di Nutrizione Umana); the compliance to the diet was monitored daily by a dietician. (2) Aerobic exercise training carried out under the guidance of a therapist. The program included two 30‐min sessions per day of cycling, treadmill walking, and stationary rowing, preceded and followed by 5–7‐min stretching, for 5 days per week with HR monitoring and medical supervision. The initial intensity of exercise was set at ~60% of HR_peak_ determined during the incremental exercise test before the intervention and was progressively increased reaching ~80% at the end of the exercise program. While RMET (see below) was performed in the morning, exercise training was administered in the afternoon. (3) Psychological and nutritional counselling.

### Respiratory muscle endurance training

An incremental RMET protocol was performed by using a commercially available device (Spiro Tiger^®^, Idiag, Fehraltorf, Switzerland), as previously described in a similar population (Rigamonti et al. 2014; Salvadego et al. 2017). The device consists of a hand‐held unit with a respiratory pouch and a base station. The specific properties of the device allow for personalized respiratory training through maximum inspirations and expirations, without hypocapnia. To avoid hypocapnia despite hyperpnea, the device features a 2‐way piston valve to rebreathing bag. As the patient breathes out through the mouthpiece, the rebreathing bag stores part of the expired air, which contain an increased CO_2_ partial pressure. Once the rebreathing bag is filled to its capacity, a valve opens allow the rest of expired air to be released to the environment. The valve shuts when expiration finishes, and inspiration starts. Inspiration empties the rebreathing bag first (filled with the exhaled air containing an increased CO_2_ partial pressure), then the valve opens, and some fresh outside air is inspired at the end of each inspiration. No symptoms of lightheadedness or malaise were described by any patient during the RMET sessions.

Personal training target values were entered into the base unit and were used to monitor breathing frequency and depth during training. The base station in the hand‐held computer monitors the breathing frequency, sets threshold limits for breathing patterns, and displays visual and acoustic feedback to allow the subject to breathe within the threshold values estimated for normocapnia.

The base station stored time and frequency of each exercise sessions, thus allowing the patient and the researcher to retrieve and review the data. The volume of rebreathing in the bag was chosen in order to obtain pulmonary ventilation ($\dot{V}$_E_) values corresponding to ~50–60% of maximal voluntary ventilation, as evaluated by spirometry before the training protocol. The duration of each RMET session increased progressively from 12 min (5 min at a respiratory frequency [fR] of 22 breaths per min, 5 min at 24 breaths per min, 2 min at 26 breaths per min) to 18 min (5 min at a fR of 24 breaths per min, 5 min at 26 breaths per min, 8 min at 28 breaths per min). In short, the patients trained for 3 weeks, 5 days per week, 1 session per day, 12–18 min per session, with a fR of 22–28 breaths per min, following an incremental protocol described in detail in Rigamonti et al. (2014).

### Spirometry

Before and after (within 2 days) the interventions (CTRL or RMET) the patients performed standard spirometry tests (forced vital capacity, FVC; forced expiratory volume in 1 sec, FEV_1_; FEV_1_/FVC; forced expiratory flow between 25% and 75% of FVC, FEF_25–75%_; maximal forced expiratory flow, FEF_max_) by utilizing a metabolic cart (MedGraphics CPX/D, Medical Graphics Corp., USA). Pulmonary function testing was performed according to the guidelines of the American Thoracic Society (Miller et al. 2005). Predicted values were based on Hankinson et al. (1999).

### Exercise protocol

Before and within 2 days after the 3 weeks of interventions (CTRL or RMET) exercise tests were conducted during 2 consecutive days under medical supervision; during the tests the subjects were continuously monitored by 12‐lead electrocardiography (ECG). A mechanically braked treadmill (TecnoGym, Italy) was utilized. Patients were allowed time to gain familiarity with the researchers and the experimental set‐up, were carefully instructed about the procedures and were familiarized with the protocol using short practice walks. Patients were asked to avoid intensive exercise for 24 h and to refrain from food and caffeine for at least 2 h before the tests. During the first day the subjects performed an incremental exercise test. After 3 min of resting measurement (subjects in standing position on the treadmill) the incremental exercise began, and the patients walked on the treadmill (0% slope) for 2 min at 3.5 km/h. The velocity was then increased by 0.5 km/h every minute till 6 km/h. When the velocity reached 5 km/h the slope was set at 3% and kept at this level till 6 km/h; thereafter the slope was increased by 0.5% every minute. When the slope reached 10.5% the velocity was increased to 6.5 km/h till the subjects reached voluntary exhaustion, defined as the inability to maintain the imposed speed and slope despite vigorous encouragement by the researchers. During the tests the patients could not hold on the handlebars of the treadmill. For all variables (see below), mean values calculated over the last 20–30 sec of the incremental exercise before reaching voluntary exhaustion were considered “peak” values.

During the second day, the patients performed one repetition of 12‐min CWR exercise at ~60% (moderate‐intensity) and at ~120% (heavy‐intensity) of the GET (see below), determined during the incremental exercise before each intervention. In order to identify the work rate corresponding to the V̇O_2_ at GET, the effect of the delayed V̇O_2_ adjustment to the increased work rate during the incremental test was corrected by shifting the linear V̇O_2_ versus time (and work rate) relationship to the left, by an amount corresponding to the individual mean response time of the V̇O_2_ kinetics determined in each subject (Whipp et al. 1981). CWR exercise at ~60% of GET was always carried out before CWR exercise at ~120% of GET. About 1 h of recovery separated the two CWR exercises. Resting V̇O_2_ (subjects in standing position on the treadmill) was measured before the CWR exercise began.

### Measurements

$\dot{V}$_E_, tidal volume (V_T_), fR, O_2_ uptake ($\dot{V}$O_2_) and CO_2_ output ($\dot{V}$CO_2_) were determined on a breath‐by‐breath basis by means of a metabolic unit (MedGraphics CPX/D, Medical Graphics Corp., USA). Calibration of O_2_ and CO_2_ analyzers was performed before each experiment by utilizing gas mixtures of known composition. Expiratory flow measurements were performed by a bidirectional pressure differential pneumotachograph, which was calibrated by a 3‐L syringe at varying flow rates. The respiratory gas‐exchange ratio (R) was calculated as $\dot{V}$CO_2_/$\dot{V}$O_2_. HR was determined by ECG. GET was determined by the V‐slope method; ventilatory equivalents ($\dot{V}$_E_/$\dot{V}$O_2_, $\dot{V}$_E_/$\dot{V}$CO_2_) were utilized as ancillary signs (Beaver et al. 1986). All the data related to GET were expressed as $\dot{V}$ O_2_ (L/min) and as a percentage of $\dot{V}$O_2peak_. Ratings of perceived exertion (RPE) for respiratory discomfort (RPE_R_) and limb effort (RPE_L_) were obtained at rest and every minute during exercise by using the Borg's modified CR10 scale (Wilson and Jones 1991).

Considering that only one repetition of each CWR exercise could be carried out, a formal $\dot{V}$O_2_ kinetics analysis was not performed (Lamarra et al. 1987). Mean $\dot{V}$O_2_ values were calculated during the last 30 sec of every minute of CWR exercises. The presence/absence of a steady state in $\dot{V}$O_2_ and HR after the first minutes of CWR exercise was evaluated by fitting linear regressions on the data obtained from the third to the last minute of exercise. Whereas the absence of a significant slope of the regression suggests a steady state, a positive slope suggests an increasing O_2_ cost of exercise as a function of time during CWR. The O_2_ cost of walking (aerobic energy expenditure per unit of covered distance) was calculated as Δ$\dot{V}$O_2_ ($\dot{V}$O_2_ determined during the task minus resting $\dot{V}$O_2_)/velocity (di Prampero et al. 2009; Lazzer et al. 2014). The O_2_ cost of walking was expressed as mL O_2_/m and as mL O_2_/kg/m.

### Statistical analysis

Results were expressed as means ± standard deviation (*x* ± SD). Considering the primary outcome of this study, the change of the O_2_ cost of walking during moderate‐ and heavy‐ intensity exercise after RMET, a sample size of 8 achieves a 90% power to detect a mean difference of 16% and a standard deviation of differences of 20% with a significance level of 0.03. This difference corresponds to the mean difference previously observed following RMET in obese adolescents during cycling (Salvadego et al. 2017). A two‐way analysis of variance (ANOVA) with repeated measures (2 groups × 2 times) was used to assess changes in anthropometry, spirometry parameters and exercise tolerance in two groups (RMET vs. CTRL) over the protocol period (pre vs. post). When a statistically significant difference was identified at ANOVA, a Bonferroni post‐hoc test was applied to locate the difference. Statistical analyses were carried out by utilizing commercially available software packages (Prism 5.0, GraphPad, USA; Statistical Package Social Sciences 15.0, SPSS Inc., USA).

## Results

The main anthropometric data are reported in Table 1. During the 3‐week hospitalization period both groups lost BM (~4–5 kg, corresponding to ~3–4% of the baseline BM); BMI and BMI‐SDS also decreased significantly after both interventions. No differences between groups at baseline were observed for these variables.

**Table 1:** Age and anthropometric characteristics of the participants in standard body weight reduction intervention (CTRL group) and standard body weight reduction intervention combined with respiratory muscle endurance training (RMET group)

	RMET group (*n *=* *8)		CTRL group (*n *=* *8)		*P* Interaction	*P* group	*P* time
	PRE	POST	PRE	POST			
Age (years)	16.5 ± 0.9	16.5 ± 0.9	15.5 ± 0.8	15.5 ± 0.8	1.00	0.03	1.00
Height (m)	1.80 ± 0.05	1.80 ± 0.05	1.75 ± 0.05	1.75 ± 0.05	1.00	0.05	1.00
Body mass (kg)	130.5 ± 18.4	125.7 ± 18.0***	124.9 ± 10.0	120.8 ± 9.4***	0.50	0.48	<0.0001
BMI (kg/m^2^)	40.4 ± 5.0	38.6 ± 5.2***	41.0 ± 2.9	39.6 ± 2.8***	0.27	0.71	<0.0001
BMI‐SDS	3.5 ± 0.6	3.3 ± 0.6***	3.7 ± 0.3	3.5 ± 0.3***	0.21	0.50	<0.0001
FFM (kg)	78.6 ± 9.3	78.7 ± 7.5	76.2 ± 5.5	74.1 ± 4.8	0.10	0.33	0.14
FM (kg)	51.9 ± 10.1	47.0 ± 10.8***	48.7 ± 4.9	46.7 ± 4.8	0.04	0.66	0.0001
FM (% of BM)	39.6 ± 3.1	36.9 ± 3.9**	38.9 ± 1.2	38.6 ± 1.3	0.04	0.69	0.01

Values are expressed as mean±SD. BMI‐SDS, SD score of body mass index (BMI); FFM, fat free mass; RMET, respiratory muscle endurance training.

***P <* *0.001; **P <* *0.01; Bonferroni post‐hoc test to locate the statistically significant differences within groups.

The main spirometry data are reported in Table 2. No restrictive or obstructive alterations were observed in both groups. FVC was significantly higher after versus before RMET, whereas no significant difference was observed following CTRL. A previous study by our group (LoMauro et al. 2016) on a similar population described that 3 weeks of RMET is enough to reduce abdominal load, recruit lung and chest wall volumes, and as a result increase FVC. No differences between groups at baseline were observed for spirometry variables.

**Table 2:** Spirometry data of patients before and after the standard intervention of body mass reduction (CTRL) and the standard intervention combined with RMET

	RMET group (*n *=* *8)		CTRL group (*n *=* *8)		*P* Interaction	*P* Group	*P* time
	PRE	POST	PRE	POST			
FVC, L	5.1 ± 0.7	5.6 ± 0.7^a^	4.4 ± 0.8	4.8 ± 0.6	0.67	0.05	0.03
FVC, % predicted	97.8 ± 12.8	106.2 ± 9.2	93.4 ± 15	101 ± 17.7	0.89	0.50	0.03
FEV_1_, L	4.3 ± 0.7	4.8 ± 0.7	3.8 ± 0.6	3.9 ± 0.4	0.19	0.06	0.10
FEV_1_, % predicted	96.5 ± 16.4	106.9 ± 13	94.1 ± 10.5	95.8 ± 6.5	0.22	0.32	0.10
FEV_1_/FVC, %	83.7 ± 4.8	85.5 ± 4.3	87 ± 6.7	82.6 ± 9.9	0.08	0.95	0.44
FEF_25–75%_	4.5 ± 0.4	5.3 ± 1.1	4.2 ± 0.8	4 ± 1	0.10	0.09	0.25
FEF_25–75%_, % predicted	92.4 ± 9	108.3 ± 23	92.4 ± 10.2	89.1 ± 16.4	0.01	0.23	0.26
PEF, L/sec	7 ± 1.2	8.5 ± 1.8	6.6 ± 0.4	7.3 ± 0.9	0.49	0.13	0.08
PEF, % predicted	74.5 ± 15.9	91.2 ± 16.1	78.7 ± 2.3	86.9 ± 8.5	0.47	0.99	0.05

FVC, forced vital capacity; FEV1, forced expiratory volume in 1 sec; FEF_25–75%_, forced expiratory flow between 25% and 75% of FVC; RMET, respiratory muscle endurance training; PEF, peak expiratory flow. Values are expressed as mean±SD.

a *P *<* *0.05; Bonferroni post‐hoc test to locate the statistically significant differences within groups.

Mean ± SD peak values of the investigated variables obtained during the incremental test are presented in Table 3. For most variables, no differences were observed after versus before both interventions, with the notable exception of the time to exhaustion and walking slope, which were significantly higher after versus before RMET, whereas no significant differences were observed after versus before CTRL. $\dot{V}$O_2_peak values, in absolute values and divided per unit of BM, are similar to those usually obtained in obese adolescents (Salvadego D et al. 2010; Hansen et al. 2014; Salvadego et al. 2015, 2017). GET, expressed as L/min of $\dot{V}$O_2_, was not affected by either intervention (2.31 ± 0.47 and 2.39 ± 0.48 in before and after RMET; 2.14 ± 0.38 and 2.04 ± 0.14 in before and after CTRL). In all conditions GET corresponded to ~70% of $\dot{V}$O_2_peak.

**Table 3:** Peak values of the main investigated variables, determined at exhaustion during the incremental exercise, before and after the standard intervention of body mass reduction (CTRL group) or the standard intervention combined with the RMET program (RMET group)

	RMET group (*n *=* *8)		CTRL group (*n *=* *8)		*P* Interaction	*P* group	*P* time
	PRE	POST	PRE	POST			
$\dot{V}$O_2_ (L/min)	3.49 ± 0.50	3.63 ± 0.58	2.91 ± 0.53	2.92 ± 0.31#	0.56	0.01	0.50
$\dot{V}$O_2_/BM (mL/min/kg)	26.6 ± 3.5	30.1 ± 7.0	23.4 ± 4.2	24.3 ± 3.3#	0.34	0.04	0.11
$\dot{V}$CO_2_ (L/min)	3.38 ± 0.60	3.63 ± 0.50	3.00 ± 0.45	2.96 ± 0.33#	0.20	0.03	0.34
fR (breaths/min)	40.2 ± 6.2	42.8 ± 7.9	43.3 ± 10.5	42.8 ± 9.3	0.21	0.72	0.36
*V*_T_ BTPS (L)	2.1 ± 0.4	2.2 ± 0.4	2.1 ± 0.3	2.1 ± 0.3	0.53	0.53	0.46
$\dot{V}$_E_ BTPS (L/min)	83.6 ± 17.0	92.9 ± 20.3	87.4 ± 22.8	86.2 ± 11.6	0.16	0.87	0.27
*R*	0.97 ± 0.06	1.01 ± 0.04	1.04 ± 0.11	1.02 ± 0.09	0.16	0.87	0.27
PETO_2_ (mmHg)	102.5 ± 6.5	103.7 ± 6.2	99.2 ± 7.2	99.3 ± 6.5	0.52	0.26	0.47
PETCO_2_ (mmHg)	45.5 ± 5.1	45.1 ± 7.5	40.2 ± 6.4	38.8 ± 5.4	0.56	0.07	0.31
$\dot{V}$_E_/$\dot{V}$O_2_	24.1 ± 3.4	25.8 ± 3.4	29.6 ± 5.2#	29.0 ± 4.1	0.04	0.04	0.31
$\dot{V}$_E_/$\dot{V}$CO_2_	24.8 ± 2.8	25.7 ± 3.5	28.5 ± 3.9	28.6 ± 3.4	0.47	0.07	0.31
HR (beats/min)	172.3 ± 15.8	173.3 ± 8.5	173.1 ± 3.8	171.7 ± 9.2	0.60	0.94	0.92
Waking velocity (km/h)	6.0 ± 0.0	6.1 ± 0.2	6.1 ± 0.2	6.1 ± 0.2	0.09	0.69	0.55
Walking slope (%)	5.9 ± 2.2	8.6 ± 2.1***	7.9 ± 2.3	8.9 ± 1.5	0.007	0.35	< 0.0001
TE (sec)	690 ± 250	1028 ± 250***	908 ± 300	1020 ± 187	0.004	0.41	< 0.0001

BM, body mass; fR, respiratory rate; HR, heart rate; PETCO_2_, CO_2_ end‐tidal pressure; PETO_2_, O_2_ end tidal pressure; *R*, respiratory gas‐exchange ratio; TE, Time to exhaustion; RMET, respiratory muscle endurance training; $\dot{V}$_E_, pulmonary ventilation; $\dot{V}$CO_2_, CO_2_ output; $\dot{V}$O_2_, O_2_ uptake; V_T_, tidal volume. Values are expressed as mean±SD.

***P <* *0.001; Bonferroni post‐hoc test to locate the statistically significant differences within groups.

^#^ *P *<* *0.05; Bonferroni post‐hoc test to locate the statistically significant differences between groups.

All patients completed the 12 min CWR protocols, both for moderate‐ and heavy‐intensity walking. Mean ± SD values of the investigated variables calculated during the last minute of the CWR protocols are reported in Table 4. During moderate‐intensity walking, RMET significantly decreased $\dot{V}$O_2_ (expressed in L/min and divided by BM), whereas no significant differences were observed in after versus before CTRL. When expressed as a percentage of $\dot{V}$O_2_peak, $\dot{V}$O_2_ values were ~49% versus ~42% in before versus after RMET, and ~49% versus ~47% in before versus after CTRL. The same trends (significant decrease after RMET, no significant difference after CTRL) were observed for $\dot{V}$_E_ and $\dot{V}$CO_2_. HR significantly (*P *<* *0.05) decreased both after RMET and after CTRL. When expressed as a percentage of HRpeak, HR values were ~67% versus ~61% before versus after RMET, and ~68% versus ~65% before versus after CTRL.

**Table 4:** Values of the main investigated variables determined during the last minute of the constant work rate (CWR) exercises carried out at 60% of the work rate of gas exchange threshold (CWR <GET) and at 120% of the work rate of GET (CWR > GET), before and after the standard body mass reduction intervention (CTRL group) and the standard intervention combined with RMET (RMET group)

CWR <GET	RMET group (*n *=* *8)		CTRL group (*n *=* *8)		*P* Interaction	*P* group	*P* time
	Pre	Post	Pre	Post			
$\dot{V}$O_2_ (L/min)	1.69 ± 0.41	1.50 ± 0.33*	1.44 ± 0.43	1.38 ± 0.54	0.17	0.39	0.01
$\dot{V}$O_2_/BM (mL/kg/min)	12.8 ± 2.3	11.6 ± 2.3	11.5 ± 2.9	11.4 ± 4.1	0.11	0.61	0.11
$\dot{V}$O_2rest_ (L/min)	0.54 ± 0.1	0.55 ± 0.09	0.41 ± 0.1	0.40 ± 0.13#	0.68	0.005	0.95
$\dot{V}$O_2rest_/BM (mL/kg/min)	4.1 ± 0.6	4.3 ± 0.6	3.3 ± 0.8	3.4 ± 1.2	0.77	0.02	0.66
$\dot{V}$CO_2_ (L/min)	1.35 ± 0.31	1.21 ± 0.35	1.20 ± 0.35	1.21 ± 0.52	0.09	0.68	0.13
fR (breaths/min)	28.0 ± 5.5	26.5 ± 4.8	30.0 ± 6.5	29.0 ± 9.0	0.90	0.41	0.55
V_T_ BTPS (L)	1.4 ± 0.4	1.3 ± 0.4	1.1 ± 0.3	1.2 ± 0.4	0.41	0.36	0.69
$\dot{V}$_E_ BTPS (L/min)	35.9 ± 8.3	32.6 ± 7.0	33.3 ± 11.4	34.6 ± 15.8	0.10	0.96	0.48
*R*	0.81 ± 0.03	0.81 ± 0.02	0.84 ± 0.05	0.88 ± 0.06##	0.25	0.01	0.16
PETO_2_ (mmHg)	95.6 ± 2.8	96.4 ± 3.3	89.7 ± 3.8#	92.4 ± 5.2	0.29	0.01	0.06
PETCO_2_ (mmHg)	43.6 ± 2.2	43.2 ± 1.8	41.8 ± 3.6	40.3 ± 3.9	0.26	0.13	0.06
$\dot{V}$_E_/*V*O_2_	21.5 ± 1.6	21.9 ± 1.6	22.5 ± 1.0	24.1 ± 1.8*	0.21	0.02	0.03
$\dot{V}$_E_/VCO_2_	26.6 ± 1.7	27.0 ± 1.5	26.8 ± 1.5	27.5 ± 2.1	0.75	0.68	0.24
HR (beats/min)	115 ± 21	105 ± 16**	117 ± 11	111 ± 14	0.36	0.60	0.0008
Walking velocity (km/h)	4.1 ± 0.5	4.1 ± 0.5	4.1 ± 0.6	4.1 ± 0.6	1.00	1.00	1.00
Walking slope (%)	0.0 ± 0.0	0.0 ± 0.0	0.0 ± 0.0	0.0 ± 0.0	1.00	1.00	1.00
CWR > GET							
$\dot{V}$O_2_ (L/min)	3.16 ± 0.56	2.89 ± 0.44**	2.62 ± 0.31#	2.45 ± 0.30	0.37	0.03	0.0008
$\dot{V}$O_2_/BM (mL/kg/min)	24.1 ± 3.0	22.6 ± 2.7*	21.1 ± 2.4	20.5 ± 2.8	0.21	0.09	0.02
$\dot{V}$O_2rest_ (L/min)	0.61.5 ± 0.17	0.57.1 ± 0.12	0.38 ± 0.12##	0.37 ± 0.08##	0.68	0.002	0.34
$\dot{V}$O_2rest_/BM (mL/kg/min)	4.6 ± 0.9	4.4 ± 0.8	3.1 ± 0.9##	3.1 ± 0.8##	0.62	0.0007	0.70
$\dot{V}$CO_2_ (L/min)	2.86 ± 0.61	2.55 ± 0.42	2.54 ± 0.28	2.47 ± 0.45	0.18	0.35	0.05
fR (breaths/min)	42.9 ± 5.2	40.0 ± 6.1	43.7 ± 10.3	41.7 ± 9.4	0.76	0.74	0.06
V_T_ BTPS (L)	1.9 ± 0.4	1.8 ± 0.5	1.8 ± 0.4	1.8 ± 0.3	0.73	0.69	0.70
$\dot{V}$_E_ BTPS (L/min)	79.2 ± 22.8	70.1 ± 16.2*	74.5 ± 12.8	71.3 ± 13.4	0.26	0.83	0.03
*R*	0.91 ± 0.04	0.88 ± 0.03	0.97 ± 0.09	1.00 ± 0.11##	0.29	0.004	0.79
PETO_2_ (mmHg)	103.9 ± 6.2	103.1 ± 6.1	98.2 ± 5.7	97.9 ± 6.5	0.81	0.12	0.66
PETCO_2_ (mmHg)	40.8 ± 4.2	41.0 ± 4.3	39.2 ± 4.7	39.7 ± 5.5	0.78	0.33	0.60
$\dot{V}$_E_/*V*O_2_	25.5 ± 3.8	24.7 ± 3.0	27.9 ± 2.6	28.1 ± 2.9#	0.43	0.02	0.70
$\dot{V}$_E_/*V*CO_2_	28.1 ± 3.3	27.9 ± 2.9	28.8 ± 2.9	28.2 ± 3.1	0.55	0.43	0.47
HR (beats/min)	160 ± 17	146 ± 18***	163.3 ± 10.6	157.7 ± 7.9	0.03	0.33	< 0.0001
RPE_R_	2.7 ± 2.8	0.8 ± 2.1*	4.3 ± 1.8	2.9 ± 1.6	0.69	0.053	0.02
RPE_L_	2.6 ± 2.7	1.2 ± 2.2	2.9 ± 2.0	3.8 ± 2.3	0.05	0.21	0.64
Walking velocity (km/h)	5.9 ± 0.2	5.9 ± 0.2	6.0 ± 0.0	6.0 ± 0.0	1.00	0.17	1.00
Walking slope (%)	4.6 ± 2.2	4.6 ± 2.2	5.3 ± 1.4	5.3 ± 1.4	1.00	0.43	1.00

BM, body mass; CWR, constant work rate; fR, respiratory rate; GET, gas exchange threshold; HR, heart rate; PETCO_2_, CO_2_ end‐tidal pressure; PETO_2_, O_2_ end tidal pressure; *R*, respiratory gas‐exchange ratio; RPE_L_, rate of perceived exertion for leg effort; RPE_R_, rate of perceived exertion for respiratory discomfort; TE, Time to exhaustion; $\dot{V}$_E_, pulmonary ventilation; $\dot{V}$CO_2_, CO_2_ output; $\dot{V}$O_2_, O_2_ uptake; *V*_T_, tidal volume; RMET, respiratory muscle endurance training. Values are expressed as mean ± SD.

***P <* *0.001, **P <* *0.01, *P <* *0.05; Bonferroni post‐hoc test to locate the statistically significant differences within groups. ^##^ *P *<* *0.01, ^#^ *P *<* *0.05; Bonferroni post‐hoc test to locate the statistically significant differences between groups.

Very similar patterns were observed for heavy‐intensity walking. RMET significantly decreased $\dot{V}$O_2_ (expressed in L/min and divided by BM), whereas no significant differences for this variable were observed in after versus before CTRL. When expressed as a percentage of $\dot{V}$O_2_peak, $\dot{V}$O_2_ values were ~92% versus ~81% in before versus after RMET, and ~92% versus ~84% in before versus after CTRL. The same trends (significant decrease after RMET, no significant difference after CTRL) were observed for $\dot{V}$E and $\dot{V}$CO_2_. HR significantly decreased after RMET but not after CTRL. When expressed as a percentage of HRpeak, HR values were ~93% versus ~84% in before versus after RMET, and ~94% versus ~91% in before versus after CTRL. No differences between groups at baseline were observed for these variables.

**Figure 1:**
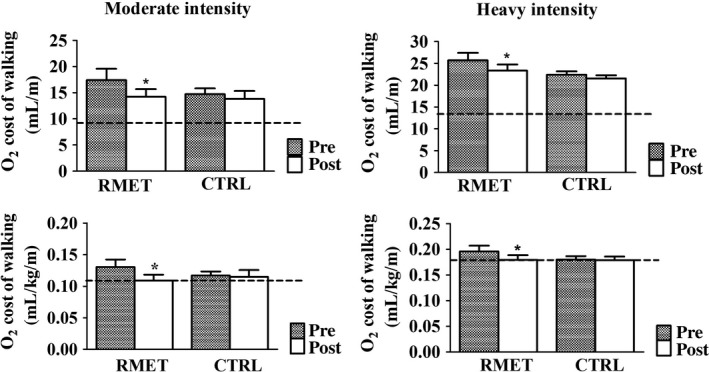
Mean (±SD) values of the O_2_ cost of walking (oxidative energy expenditure per unit of covered distance, calculated as Δ$\dot{V}$O_2_ per velocity) during the last minute of CWR exercise at ~60% of GET (moderate‐intensity, left panels) and at ~120% of GET (heavy‐intensity, right panels), before and after CTRL and RMET. In the upper panels the O_2_ cost of walking is expressed as mL O_2_/m, whereas in the lower panels the variable is normalized per unit of BM (mL O_2_/kg/m). Dashed horizontal lines are reference values from the literature (Margaria et al. 1963; di Prampero 1986; Ekelund et al. 2004) for a man with a BM of 75 kg. RMET reduced significantly the O_2_ cost of walking. See text for further details. *P <* *0.05. Bonferroni post‐hoc tests to locate the statistically significant difference (after vs. before RMET). BM, body mass; CWR, constant work rate; GET, gas exchange threshold; RMET, respiratory muscle endurance training.

Mean (±SD) values of the O_2_ cost are presented for moderate‐intensity (left panels) and heavy‐intensity (right panels) walking in Figure 1. Data are expressed as mL O_2_/m (upper panels) and as mL O_2_/kg/m (lower panels). In all experimental conditions values calculated during heavy‐intensity walking were higher than those obtained during moderate‐intensity walking. For both exercise intensities, the O_2_ cost of walking decreased following RMET, but not following CTRL. For moderate and heavy‐intensity walking, reference values from the literature (Margaria et al. 1963; di Prampero 1986; Ekelund et al. 2004) for a man with a BM of 75 kg are also shown in the figure (dashed horizontal line). The data of the obese patients from the present study are more than 100% higher than the reference value when expressed as mL O_2_/m, whereas the difference becomes much smaller (before RMET) or substantially disappears (after RMET) when the O_2_ cost of walking is normalized by BM.

**Figure 2:**
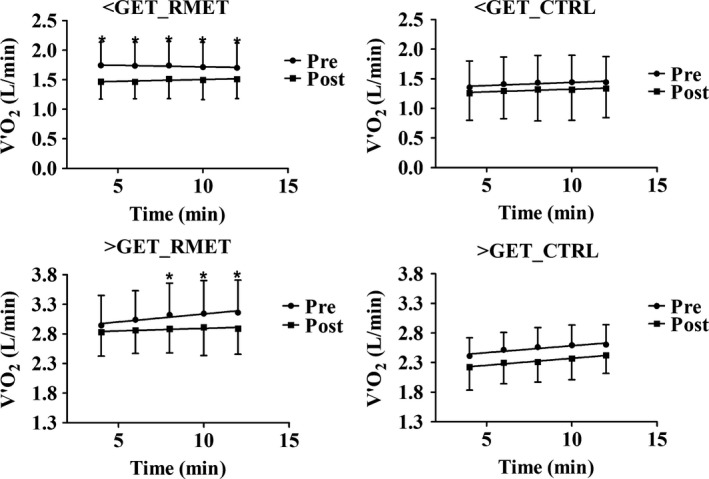
Mean (±SD) $\dot{V}$O_2_ values calculated every 2 min, from the third to the last minute of CWR walking at ~60% of GET (moderate‐intensity, upper panels) and at ~120% of GET (heavy‐intensity, lower panels), before and after RMET and CTRL. During CWR <GET mean values of the individual slopes of the linear regressions of $\dot{V}$O_2_ versus time were not significantly different from zero, before and after both interventions. At all‐time points $\dot{V}$O_2_ values were significantly lower after versus before RMET, whereas no statistically significant differences were observed after versus before CTRL. During CWR >GET the slopes of the linear regressions of $\dot{V}$O_2_ versus time were significantly greater than zero before both RMET and CTRL. The mean values of the individual slopes were significantly lower after versus before RMET, but not after versus before CTRL. See text for further details. *P <* *0.05. CWR, constant work rate; GET, gas exchange threshold; RMET, respiratory muscle endurance training.

Mean (±SD) $\dot{V}$O_2_ values calculated every 2 min, from the third to the 12th minute of exercise, during moderate‐intensity walking (upper panels) and heavy‐intensity walking (lower panels), are shown in Figure 2. All data were fitted by linear regression lines, which are shown in the Figure. During moderate‐intensity walking the mean values of the individual slopes of the linear regressions of $\dot{V}$O_2_ versus time were not significantly different from zero, before (−0.007 ± 0.010 and 0.010 ± 0.012 L/min^2^ in RMET and CTRL, respectively) and after (0.006 ± 0.010 and 0.009 ± 0.013 L/min^2^) both interventions. In other words, in all cases $\dot{V}$O_2_ was in a condition of steady state. At all‐time points, $\dot{V}$O_2_ values were significantly lower after versus before RMET, whereas no statistically significant differences were observed after versus before CTRL.

During heavy‐intensity walking the slopes of the linear regressions of $\dot{V}$O_2_ versus time were significantly greater than zero before both RMET and CTRL. In other words, before both interventions $\dot{V}$O_2_ was not in a condition of steady‐state but kept increasing from the third to the last minute of exercise. The mean values of the individual slopes were significantly lower after (0.009 ± 0.015 L/min^2^) versus before (0.027 ± 0.011) RMET, but not after (0.024 ± 0.022 L/min^2^) versus before CTRL (0.030 ± 0.014). The amplitude of the $\dot{V}$O_2_ (in L/min) increase between the third and the last minute of exercise was significantly lower after (0.06 ± 0.11) versus before (0.22 ± 0.08) RMET, but not after (0.20 ± 0.19) versus before (0.21 ± 0.08) CTRL. The same patterns were described when $\dot{V}$O_2_ values were divided by BM (data not shown).

**Figure 3:**
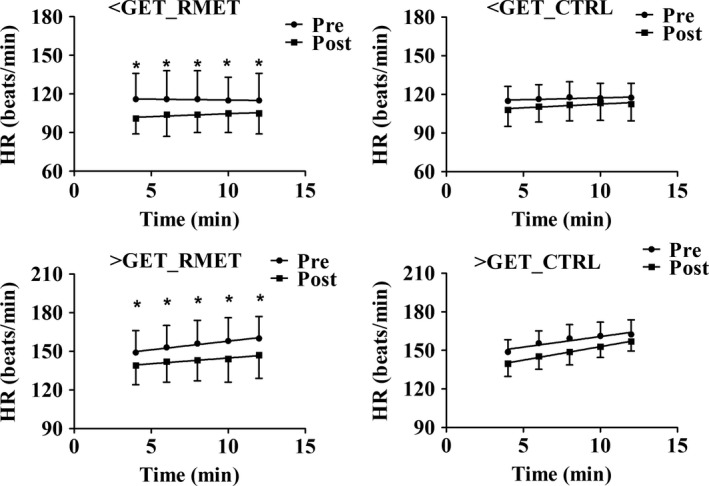
Mean (±SD) HR values calculated every 2 min, from the third to the last minute of CWR walking at ~60% of GET (moderate‐intensity, upper panels) and at ~120% of GET (heavy‐intensity, lower panels), before and after RMET and CTRL. During CWR <GET (upper panels) the mean values of the individual slopes of the linear regressions of HR versus time were not different from zero, before and after both interventions. At all time‐points values after RMET were significantly lower than those before RMET; no significant differences were observed in after versus before CTRL. During CWR >GET (lower panels) the slopes of the linear regressions of HR versus time were significantly greater than zero before both RMET and CTRL. The mean values of the individual slopes were significantly lower after versus before RMET, but not after versus before CTRL. See text for further details. *P <* *0.05. CWR, constant work rate; GET, gas exchange threshold; RMET, respiratory muscle endurance training.

The same analyses carried out for $\dot{V}$O_2_ in Figure 2 were carried out for HR in Figure 3. The results were substantially the same. During moderate‐intensity walking (upper panels) the mean values of the individual slopes of the linear regressions of HR versus time were not different from zero, before (−0.23 ± 0.48 and 0.3 ± 0.4 beats per min^2^ in RMET and CTRL, respectively) and after (0.46 ± 0.45 and 0.59 ± 0.47 beats per min^2^) both interventions. In other words, HR was in a condition of steady state. At all time‐points values after RMET were significantly lower than those before RMET; no significant differences were observed in after versus before CTRL.

During heavy‐intensity walking (lower panels) the slopes of the linear regressions of HR versus time were significantly greater than zero before both RMET and CTRL. The mean values of the individual slopes were significantly lower after (0.88 ± 0.61 beats per min^2^) versus before (1.43 ± 0.56) RMET, but not after (2.10 ± 1.26 beats per min^2^) versus before CTRL (2.19 ± 1.33).

**Figure 4:**
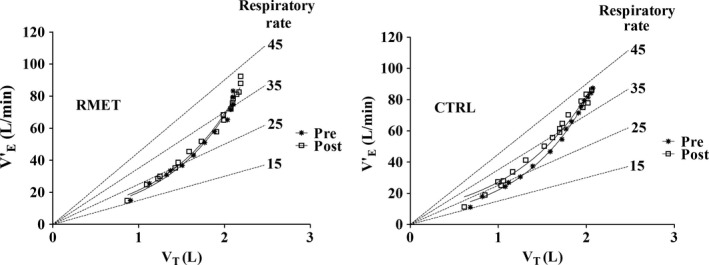
Pattern of breathing in two group of subjects during the incremental test. The relationships between mean values of pulmonary ventilation ($\dot{V}$_E_) and tidal volume (V_T_) for RMET (left panel) and CTRL (right panel) are presented, before and after the interventions. Iso‐respiratory frequency (fR) lines (dashed lines, departing from the origin) are also presented. The exponential functions fitting the experimental points are shown. See text for further details. RMET, respiratory muscle endurance training.

In order to check if the effects of RMET were associated with changes in the ventilatory pattern (the relative contribution of V_T_ and fR increases to the $\dot{V}$_E_ increase), the latter was specifically investigated by the analysis depicted in Figure 4 (mean values obtained in the different groups during incremental exercise). In the Figure, $\dot{V}$_E_ data were plotted as a function of V_T_, and iso‐fR lines were also drawn. The breathing pattern was not affected by either intervention. The rather small (but statistically significant) $\dot{V}$_E_ peak increase following RMET was attributable to an increased fR.

## Discussion

In obese male adolescents, a relatively short (3 weeks) program of RMET (Rigamonti et al. 2014), superimposed on a standard multidisciplinary BM reduction intervention (moderate caloric restriction, aerobic exercise training, psychological, and nutritional counselling) significantly reduced the O_2_ cost of walking and ameliorated signs of exercise tolerance (increased time to exhaustion during an incremental test, lower HR and RPE for the same walking velocity). The improvements were not observed in the control group of patients (CTRL), which underwent only to the multidisciplinary BM reduction intervention.

### Decreased O_2_ cost of walking

In the present study the O_2_ cost (oxidative energy expenditure per unit of distance) of moderate intensity walking was more than 100% higher in obese adolescents compared to that usually observed in normal controls. This difference is higher than that (about 50%) usually observed for cycling (Wasserman and Whipp 1975; Lafortuna et al. 2008). As mentioned in the Introduction, a higher O_2_ cost of exercise is inevitably associated with an impaired exercise tolerance (Grassi et al. 2015). It is not surprising, then, that in the present study the decreased O_2_ cost of walking observed following RMET improved exercise tolerance.

The positive effects of RMET on the O_2_ cost of walking were observed both during moderate‐ and heavy‐intensity walking on a treadmill. On the other hand, in a previous study by our group (Salvadego et al. 2017), carried out on obese adolescents exercising on a cycle ergometer, the positive effects of RMET on the O_2_ cost of exercise were observed only during heavy‐intensity CWR exercise. The results suggest that during weight bearing activities like walking or running, in which larger muscle masses are involved compared to cycling, and the patient undergoes cyclical elevations and accelerations of the body's center of mass at every step, obese adolescents are penalized, from the O_2_ cost of exercise point of view, also during moderate‐intensity exercise.

The reduced O_2_ cost of walking following RMET was exemplified by the lower $\dot{V}$O_2_ levels (during moderate‐ and heavy‐intensity walking) and by the lower $\dot{V}$O_2_ versus time slopes (during heavy‐intensity walking) (Fig. 2). No formal analyses of the $\dot{V}$O_2_ kinetics and its different components (Jones et al. 2011) was carried out in the present study, since the patients could perform only one repetition of each exercise. Some data, however, allow us to hypothesize, with reasonable confidence, that the reduced O_2_ cost during heavy intensity walking was likely attributable to a reduced amplitude or to the disappearance of the “slow component” of the $\dot{V}$O_2_ kinetics (Jones et al. 2011). The slope of the linear increase of the $\dot{V}$O_2_ versus time relationship (“excess VO_2_,” characteristic of the $\dot{V}$O_2_ slow component [(Grassi et al. 2015)]), determined from the third to the 1ast minute of exercise, was indeed substantially decreased following RMET, whereas it was not affected by CTRL. A smaller amplitude of the slow component of the $\dot{V}$O_2_ kinetics is intrinsically associated with less inefficiency and less fatigue (Jones et al. 2011; Grassi et al. 2015).

The lower O_2_ cost of walking observed in the present study after RMET could be due to a lower O_2_ cost of breathing, to a lower O_2_ cost of work by locomotor muscles or to both factors. A limitation of the present study is represented by the fact that no direct measurements of the work of breathing and of the O_2_ cost of breathing were performed. In a previous study carried out by our group in obese adolescents during cycling, however, we observed by optoelectronic plethysmography significant changes of static and dynamic thoraco‐abdominal volumes of breathing following a RMET protocol identical to that of the present study (LoMauro et al. 2016). More specifically, the patients showed abdominal rib cage hyperinflation as a form of lung recruitment during exercise, with a move to higher operating volumes. Three weeks of RMET were enough to reduce the abdominal load, recruit lung and chest wall volumes, unload RM and delay the abdominal rib cage hyperinflation (LoMauro et al. 2016). These effects, which were not determined in the present study, resulted in a reduced dyspnea and an enhanced exercise tolerance, and would be presumably associated with reduced work of breathing and O_2_ cost of breathing.

In the present study RMET did not affect the ventilatory pattern, differently from what observed by Passoni et al. (2015) in overweight patients affected by the metabolic syndrome undergoing a period of exercise training. In these patients a decreased V_T_ (and therefore a higher fR) for the same $\dot{V}$E was indeed observed after training, and the authors interpreted the data as a less costly ventilatory pattern (Passoni et al. 2015). Although in the present study the ventilatory pattern was not affected by RMET, the $\dot{V}$_E_ peak increase following RMET was attributable to an increased fR.

With some calculations, we tried to partition the relative roles of a reduced O_2_ cost of breathing and of a reduced O_2_ cost of locomotor muscles on the observed decrease of the O_2_ cost of walking. In the present study RMET lowered the $\dot{V}$O_2_ by ~190 mL/min during moderate‐intensity exercise and by ~280 mL/min during heavy‐intensity exercise. The effects of CTRL on this variable were, respectively, ~60 and ~180 mL/min. In order to estimate the $\dot{V}$O_2_ of RM ($\dot{V}$O_2_), we utilized the equations proposed by Coast et al. (1993) for normal subjects, relating $\dot{V}$E to the work of breathing, and the work of breathing to RM $\dot{V}$O_2_. Since the equations proposed by Coast et al. (1993) were obtained in normal subjects, in order to apply them to the obese population the work of breathing was increased by 70%, as proposed by Koenig (2001). After doing so, RMET decreased RM $\dot{V}$O_2_ by 7 mL/min during moderate‐intensity exercise, and by 40 mL/min during heavy‐intensity exercise. Substantially no changes were observed following CTRL. The decreases in RM $\dot{V}$O_2_ following RMET corresponded to ~4% of the decrease in $\dot{V}$O_2_ during moderate‐intensity exercise, and to ~14% during heavy‐intensity exercise. In other words, a vast majority of the O_2_ cost of walking decrease following RMET can be attributed to the decreased O_2_ cost of locomotor muscles, particularly during moderate‐intensity exercise. Although the calculations described above are based upon some assumptions and may be somewhat imprecise, the “size” of the phenomenon (85–95% of the reduced O_2_ cost of walking likely not directly attributable to a reduced O_2_ cost of breathing) should make the concept relatively “safe.”

The reduced O_2_ cost by locomotor muscles, however, might as well be a consequence of the effects of RMET on RM. By improving respiratory muscle function, indeed, RMET could prevent, reduce or delay the development of fatigue within these muscles and the reflex vasoconstriction within the active locomotor muscles (Witt et al. 2007; Romer and Polkey 2008), allowing for a greater muscle O_2_ availability. Above a threshold for respiratory muscle work, accumulation of fatigue‐related metabolites in RM could stimulate group III and IV afferent fibers going to cardiorespiratory control centers, enhancing effort perception (dyspnea) (Amann et al. 2010), determining a sympathetically‐mediated vasoconstriction of locomotor muscles, leading to decreased efficiency and fatigue (Grassi et al. 2015), and presumably also to an inhibition of central motor output (Gandevia 2001). It has been demonstrated that respiratory muscle training increases the intensity of the inspiratory muscle work necessary to activate this reflex (Witt et al. 2007).

In normal subjects a “competition” between respiratory and locomotor muscle for the finite cardiac output and the finite capacity of cardiovascular O_2_ delivery (Dempsey et al. 2006) appears to be critical during maximal or near maximal exercise (Dominelli et al. 2017), but not during submaximal tasks (Wetter et al. 1999). However, after considering the significantly increased respiratory muscle work and O_2_ cost associated with obesity (Koenig 2001), as well as the increased stress on the cardiovascular system imposed by the greater BM, it appears reasonable to hypothesize that such competition could manifest in obese patients also during submaximal tasks. The problem could be even more significant during treadmill exercise, because of the larger skeletal muscle mass involved in the task and of the weight bearing work. It should also be remembered that, by the same mechanisms, RMET could also positively affect “central” hemodynamics (Vogiatzis et al. 2011; Louvaris et al. 2014) and further enhance O_2_ delivery to the locomotor muscles. An increased peripheral O_2_ delivery would delay the development of inefficiency and fatigue within these muscles (Hogan et al. 1999; Jones et al. 2011; Grassi et al. 2015) and would reduce the amplitude of the $\dot{V}$O_2_ slow component.

The results of the present study and of the previous one (Salvadego et al. 2017) should be interpreted in conjunction with another recent study carried by our group in obese adolescents (Salvadego et al. 2015), in which acute respiratory muscle unloading, obtained by normoxic helium breathing, reduced the O_2_ cost of cycling and the perception of fatigue during moderate‐ and heavy‐intensity CWR exercise. Taken together, these studies point to the respiratory system as a target for interventions aimed at interrupting the vicious cycle between physical inactivity and obesity.

The effectiveness of RMET on 18–50 years‐old obese patients had been previously suggested by Frank et al. (2011), who observed that 7 months of RMET reduced the sensation of breathlessness during exercise and obtained an increase in the distance covered during a 12‐min time trial. These authors, however, did not perform a formal evaluation of exercise capacity and tolerance, and could not identify mechanisms potentially responsible for the enhanced performance. These limitations were overcome in our previous study (Salvadego et al. 2017) and in the present one, in which several physiological variables related to exercise tolerance were directly determined, allowing mechanistic insights into the factors potentially responsible for the observed changes, such as the effects of RMET on respiratory mechanics (LoMauro et al. 2016), the reduced O_2_ cost of exercise, the “metaboreflex” concept discussed above. The same concepts apply to another study (Edwards et al. 2016), in which a different type of respiratory muscle training (inspiratory muscle strength training) was utilized.

### Enhanced exercise tolerance

RMET increased exercise tolerance, as demonstrated by the increased time to exhaustion during the incremental test, as well as by the lower HR and RPE (during heavy intensity CWR exercise) for the same walking velocity. Reduced RPE following RMET was described also by LoMauro et al. (2016). Further linking the reduced O_2_ cost of walking to an enhanced exercise tolerance, in the present study the effects of RMET on $\dot{V}$O_2_ (Fig. 2) were substantially identical to those described for HR (Fig. 3): lower values of both variables during moderate‐ and heavy‐intensity walking, less pronounced progressive increases of both variables during heavy‐intensity walking. Textbook physiology states that, for the same work rate, lower HR corresponds to increased exercise tolerance.

The reduced O_2_ cost of exercise is likely responsible for the increased time to exhaustion during the incremental test, despite the absence of changes of $\dot{V}$O_2_peak. An increased peak work capacity, in the presence of an unchanged $\dot{V}$O_2_peak, can indeed be explained by an increased efficiency (or decreased inefficiency) of work (Lazzer et al. 2014). The lack of effects of RMET on $\dot{V}$O_2_peak is consistent with previous observations in healthy normal‐weight subjects (Edwards and Cooke 2004; Esposito et al. 2010), as well as with the results of our previous study in obese adolescents during cycling (Salvadego et al. 2017).

### Study limitations

The O_2_ cost of walking before the interventions was lower in CTRL than in RMET, both during CWR <GET, CWR >GET and at VO_2_peak. No clear‐cut explanations can be forwarded for this unexpected finding. The two groups of patients were selected a few months apart, but their age, sex and BM were not statistically significantly different. Clinical conditions, inclusion and exclusion criteria were the same. Adherence to the treatments was the same, and the patients underwent the same dietary intervention. The instruments utilized during the measurements, technical personnel and researchers were the same. No patient was allowed to hold on the handlebars of the treadmill during the tests. In principle, it cannot be excluded that in CTRL the lack of effects of the intervention on the O_2_ cost of walking could be attributed, at least in part, to the relatively low O_2_ cost in this group before the intervention. Nonetheless, the main message of the study appears to be safe: RMET significantly decreased the O_2_ cost of walking in obese adolescents, both during moderate‐ and heavy‐exercise, and it improved exercise tolerance.

The efficacy of RMET and of other types of respiratory muscle training on exercise tolerance and performance in healthy subjects, athletes and patients represents a controversial issue. Evidence in favour (McConnell 2012) or against (Patel et al. 2012) such efficacy has been recently presented in “Crosstalk” articles. In the present study, as well as in our previous one (Salvadego et al. 2017) carried out in obese adolescents, we provide objective evidence of efficacy, in terms of a reduced inefficiency of oxidative metabolism, which was associated with objective evidence of enhanced exercise tolerance.

Other potential limitations of the present study (only adolescents studied, no females, absence of a supramaximal validation test for $\dot{V}$O_2_peak determination (Poole and Jones 2017), relatively short duration of the training intervention) have been discussed in a recent study by our group in a similar group of patients (Salvadego et al. 2010).

## Conclusions

The present study, carried out on obese adolescents walking on a treadmill, extends the positive effects of a short (3‐week) RMET program on the O_2_ cost of exercise and on exercise tolerance, previously described by our group in obese adolescents only during heavy‐intensity cycling exercise (Salvadego et al. 2017), also during moderate‐intensity exercise. This appears to be relevant in terms of exercise tolerance and quality of life, since most activities of everyday life are mainly of moderate intensity. As it could be expected, in obese adolescents, respiratory limitations negatively impact on the O_2_ cost of walking and on exercise tolerance more markedly when treadmill walking is involved, compared to cycling exercise. By contrasting the vicious circle of obesity → early fatigue → reduced exercise tolerance → reduced physical activity → obesity, the intervention could represent a useful adjunct in the control of obesity. Longer periods of RMET should be investigated.

## Conflicts of Interest

No conflicts of interest, financial or otherwise, are declared by the authors.
